# Pattern interaction effect

**DOI:** 10.1038/s41598-021-93707-6

**Published:** 2021-07-16

**Authors:** J. M. Floryan, A. Inasawa

**Affiliations:** 1 Department of Mechanical and Materials Engineering, The University of Western Ontario, London, ON N6A 5B9 Canada; 2 Department of Aeronautics and Astronautics, Tokyo Metropolitan University, Asahigaoka 6-6, Hino, Tokyo, 191-0065 Japan

**Keywords:** Applied physics, Fluid dynamics

## Abstract

Unexpected responses of physical systems to external stimuli can be observed when the stimuli are organized into spatial patterns and, especially, when stimuli of different physical origins are involved, leading to the pattern interaction problem. Combinations of weak stimuli—individually only capable of producing marginal local responses—can produce a global response without involving any bifurcations. Its existence is demonstrated by the interaction of properly tuned topography and temperature patterns. When these patterns overlap in a symmetry preserving manner, the resulting convection has the form of local rolls. When these patterns are misaligned, the resulting convection involves global horizontal particle movement with direction depending on the type of misalignment.

## Introduction

The existing theories ^1^ are unable to account for the pattern interaction effect. To demonstrate the existence of this effect, we select a reference configuration consisting of a horizontal slot formed by two smooth isothermal plates with the lower plate kept at a higher temperature than the upper plate. This is a classical problem where the system response involves pure conduction if the temperature difference between the plates is small enough and convection when this difference exceeds a critical level, resulting in cellular motion known as Rayleigh-Bénard convection ^2,3^. The critical conditions are expressed in terms of the uniform Rayleigh number ${Ra}_{uni}$ whose definition together with definitions of all other parameters are given in the “Methodology” section. This configuration is spatially invariant under subcritical conditions.

**Figure 1:**
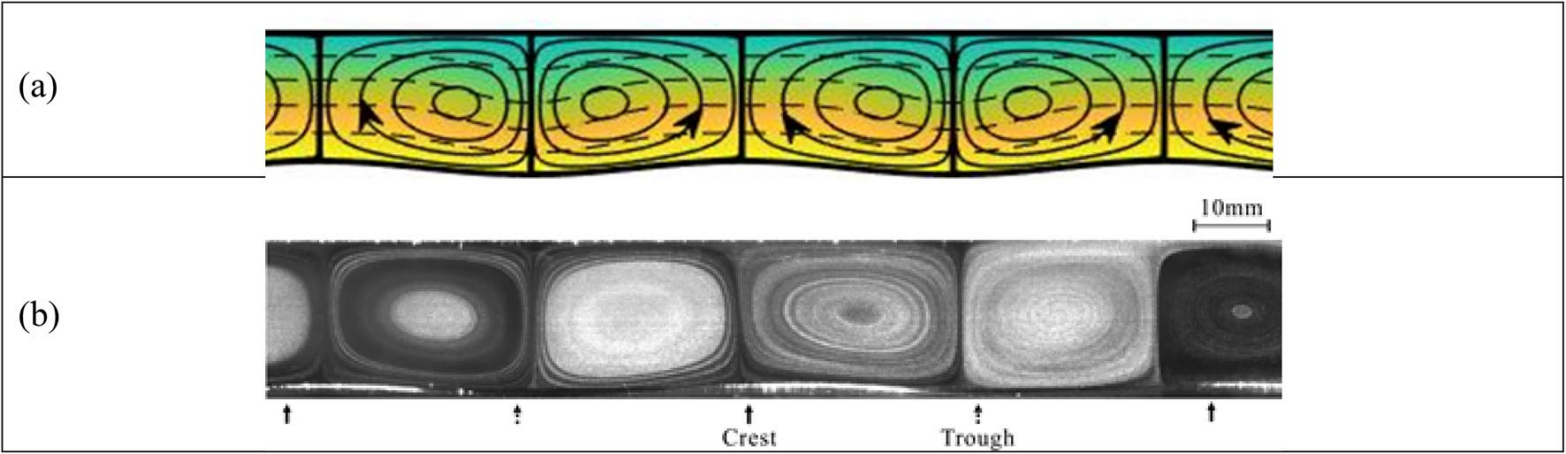
Convection in a slot formed by isothermal plates, with spatial modulations entering the system in the form of periodic grooves added to the lower plate for uniform Rayleigh number ${Ra}_{uni}=210$, the groove amplitude *A* = 0.1 and the groove wave number $\alpha =1$. Further details are provided in the “Methodology” section. (**a**) Theory (colors illustrate the temperature field with equally spaced isotherms; blue—the lowest temperature); (**b**) experiment (different levels of grey result from smoke visualization; Supplementary movie A).

The above system is altered using external stimuli of two different physical origins: (1) spatial topography patterns; and (2) spatial heating patterns, with the thermal conditions selected to preclude bifurcations to secondary states ^4^. At first, both stimuli are applied individually. The first stimulus consists of spatial modulation created by a pattern of two-dimensional grooves added to the lower plate while keeping it isothermal. In the experiment, these grooves have a sinusoidal form. The theoretical and experimental results presented in Fig. 1 demonstrate that the response involves convection with the fluid moving upwards above the groove crests and downwards above the groove troughs, forming local counter-rotating rolls, with the topology of the rolls being dictated by the surface topography. Details of the theoretical modeling and experimental set up are provided in the “Methodology” section.

**Figure 2:**
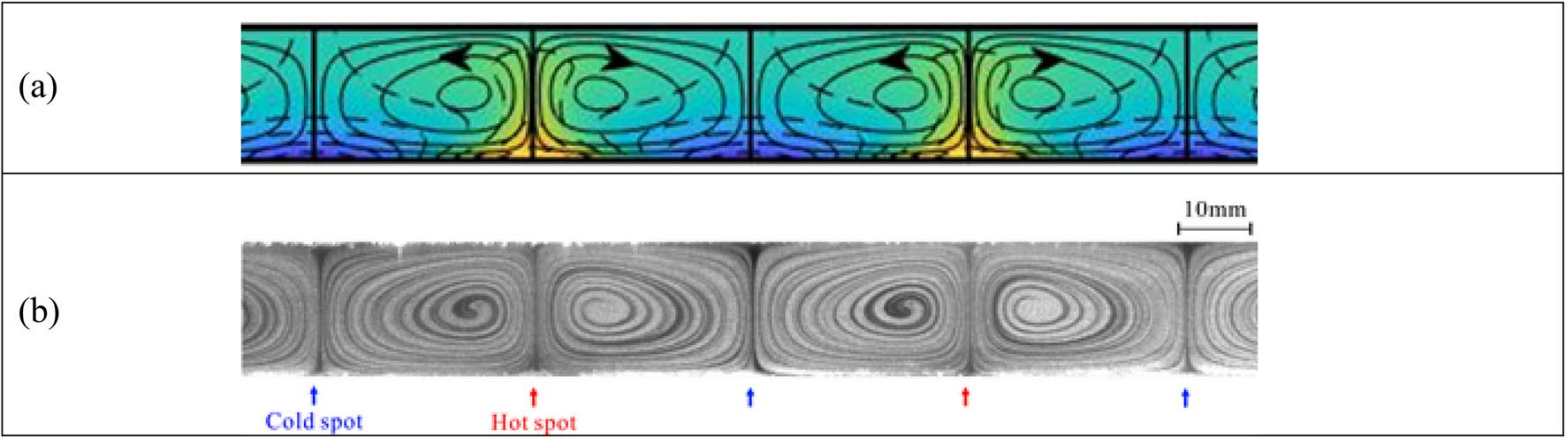
Convection in a slot formed by smooth plates, with spatial modulations entering the system in the form of periodic heating applied at the lower plate while the upper plate is kept isothermal for the periodic Rayleigh number ${Ra}_{p}=1500$ and the heating wave number $\alpha =1$. Further details are provided in the “Methodology” section. (**a**) Theory (colors illustrate the temperature field with equally spaced isotherms; blue—the lowest temperature); (**b**) experiment (different levels of grey result from smoke visualization; Supplementary movie B).

The effect of the second external stimulus is demonstrated using smooth plates and applying an external periodic heating pattern to the lower plate. The theoretical and experimental results presented in Fig. 2 demonstrate that the system response consists of local counter-rotating convection rolls with the fluid moving upwards above the hot spots (local temperature maxima) and downwards above the cold spots (local temperature minima), with the topology of the flow field being dictated by the heating pattern.

**Figure 3:**
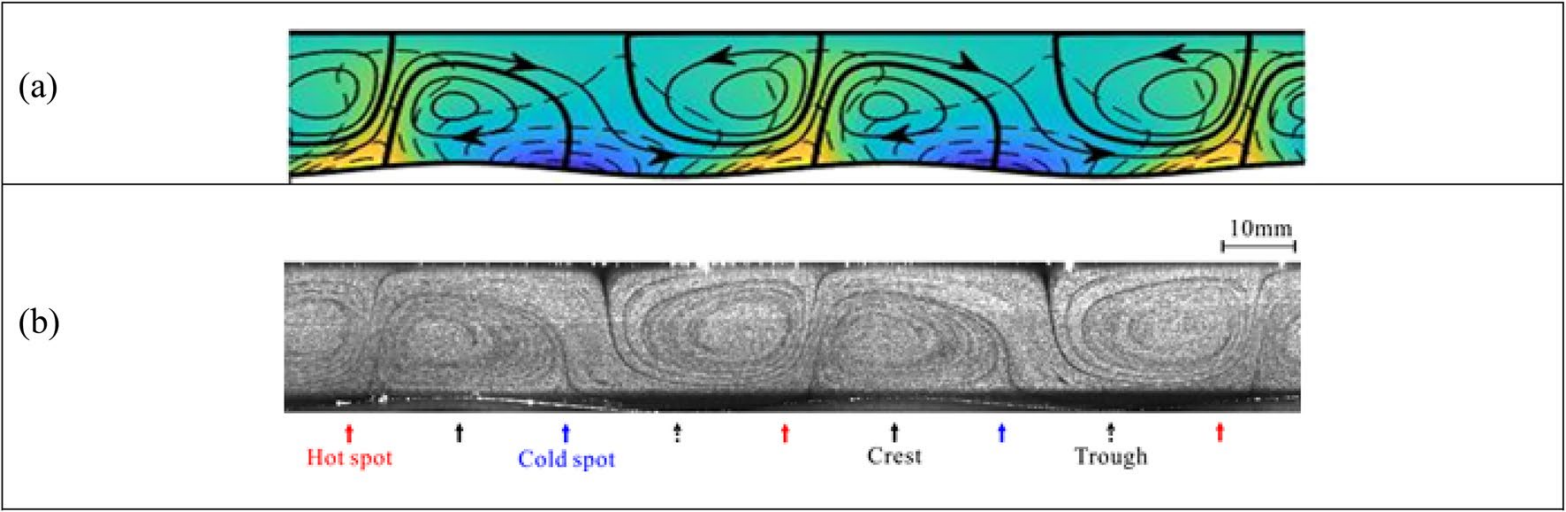
Convection in a slot in the presence of two types of spatial modulations, i.e., periodic grooves and periodic heating. Grooves have amplitude $A=0.1$ and heating has amplitude corresponding to the periodic Rayleigh number ${Ra}_{p}=1500$. Spatial distributions of both stimuli are characterized by the same wave number $\alpha =1$ (perfect tuning) and their relative position is described by the phase difference $\Omega =\mathrm{\pi }/2$. Further details are provided in the “Methodology” section. (**a**) Theory (colors illustrate temperature field with equally spaced isotherms; blue—the lowest temperature); (**b**) experiment (different levels of grey result from smoke visualization; see Supplementary movie C1).

**Figure 4:**
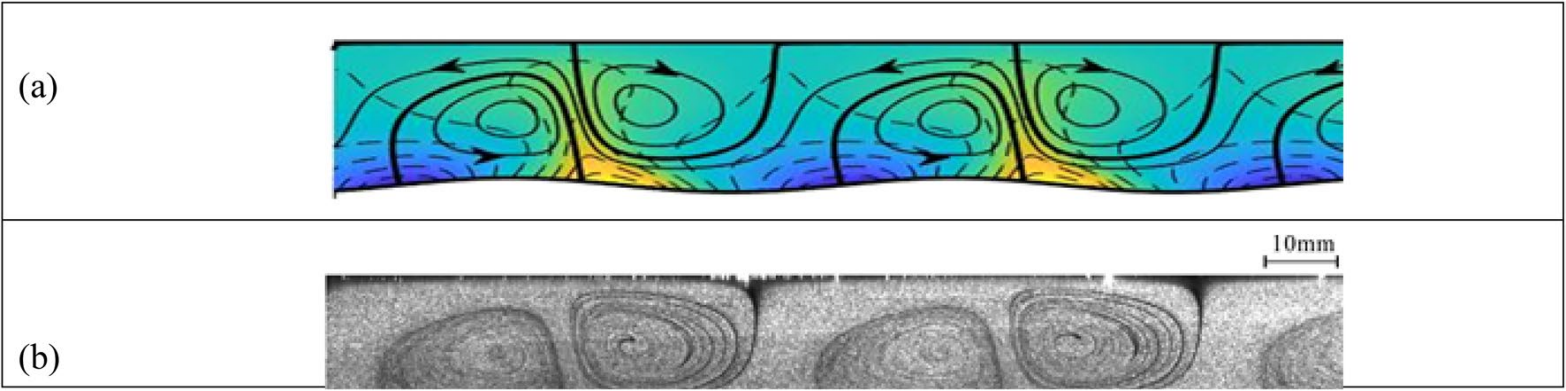
Convection for the same conditions as in Fig. 3 but with the position of the heating pattern characterized by the phase difference $\Omega =-\pi /2$. (**a**) Theory; (**b**) experiment (Supplementary movie C2).

**Figure 5:**
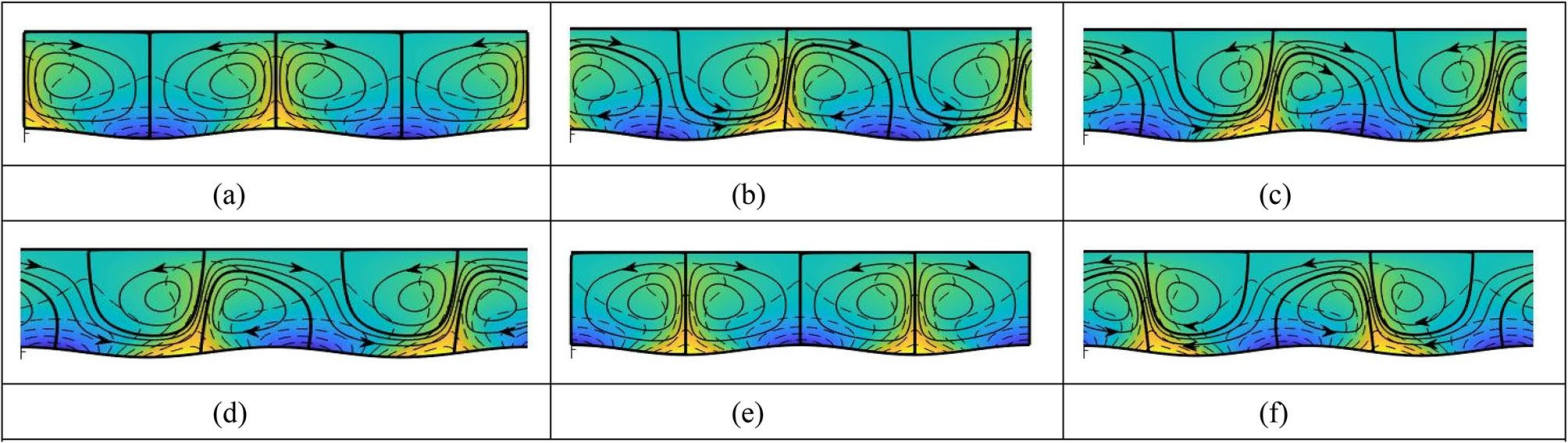
Flow patterns for the same geometry as in Figs. 3 and 4 for the phase difference $\Omega =0,\mathrm{\pi }/4,\mathrm{\pi }/\mathrm{2,3}\mathrm{\pi }/4,\mathrm{\pi },5\mathrm{\pi }/4$ in (**a**–**f**). The lower plate temperature is expressed by a single Fourier mode with the amplitude corresponding to the periodic Rayleigh number ${Ra}_{p}=1500$ and the mean resulting in the uniform Rayleigh number ${Ra}_{uni}=0.$ Colors illustrate the temperature field with equally spaced isotherms (blue corresponds to the lowest temperature).

The system’s response to the simultaneous presence of both external stimuli is more complex as it depends on the tuning and relative position of both patterns. To demonstrate the pattern interaction effect, we select perfectly tuned stimuli, i.e., the heating and groove patterns are characterized by the same wave number. The relative position of both patterns is quantified by the phase shift $\Omega$ varying in the range $\left(-\pi ,+\pi \right)$. When $\Omega =0$, the hot spots are aligned with the groove crests while for $\Omega =\mathrm{\pi }$ (or $\Omega =-\mathrm{\pi }$) the hot spots are aligned with the groove troughs, resulting in certain symmetries. Any other value of $\Omega$ breaks the symmetries, leading to a global response. In the experiment, grooves have a sinusoidal form while the measured spatial distribution of the lower plate’s temperature requires several Fourier modes for its characterization. Figure 3 demonstrates that the system’s response for $\Omega =\pi /2$ involves a combination of counter-rotating rolls and a stream tube carrying fluid in the positive *x*-direction. The formation of this stream tube demonstrates the global character of the response as it permits infinite horizontal translation of fluid elements. When $\Omega =-\pi /2$, the flow direction in the stream tube is reversed, as documented both experimentally and theoretically in Fig. 4. Theoretical results presented in Fig. 5 illustrate in a systematic manner the evolution of the system response as $\Omega$ varies from $0$ to $5\pi /4$, from movement consisting of just local rolls through different combinations of rolls and stream tubes with gradually increasing and then decreasing mean flow rate, back to just the rolls and the formation of stream tubes carrying fluid in the opposite direction. When the hot spots overlap either with the groove crests ($\Omega =0$, Fig. 5a) or with the groove troughs ($\Omega =\pi$, Fig. 5e), the flow and temperature fields exhibit symmetries. Misalignment of the grooves and temperature patterns destroys these symmetries, resulting in a global response (Fig. 5b–d,f).

## Summary

It has been shown that misalignments of spatial patterns of external stimuli results in qualitatively different system responses whose character changes from local to global depending on the magnitude of the misalignment. We refer to phenomena resulting from the misalignment of patterns as the pattern interaction effect. In the case study used to demonstrate this effect, perfectly tuned spatial patterns of external heating and topography were used. It has been shown that each stimulus acting individually was able to create only a local response with fluid particles moving within individual convection cells. On the contrary, both stimuli acting simultaneously led to a global response permitting infinite horizontal translations of fluid particles. The presence of weak stimuli can be difficult to observe in nature while the easily observable global response can be difficult to explain without invoking the concept of pattern interaction.

## Methodology—theory

The horizontal slot has a smooth upper plate and sinusoidal grooves along the lower plate with its geometry described by

1 $$y_{L} = - 1 + A\;cos\left( {\alpha x} \right),y_{U} = 1$$

where *A* is the groove amplitude and $\alpha$ is the wave number, and subscripts *L* and *U* refer to the lower and upper plates, respectively. This slot is exposed to an external heating resulting in the plates’ temperatures of the form

2 $${\theta }_{L}\left(x\right)={{Ra}_{uni}+Ra}_{p}\sum _{n=-\infty ,n\ne 0}^{n=+\infty }{{\theta }_{p}^{\left(n\right)}e}^{in\left(\alpha x+\Omega \right)},{\theta }_{U}\left(x\right)=0.$$

here, ${Ra}_{uni}=g\Gamma {h}^{3}{\theta }_{uni}/(\kappa \nu )$ and ${Ra}_{p}=g\Gamma {h}^{3}{\theta }_{p}/(\kappa \nu )$ are the uniform and periodic Rayleigh numbers with the former measuring the magnitude of the vertical temperature gradient while the latter measures the magnitude of the horizontal temperature gradient. The relative temperature $\theta$ is defined as $\theta = T{-}T_{U}$ where *T* stands for the temperature and ${T}_{U}=const$ denotes the temperature of the upper isothermal plate, ${\theta }_{uni}={T}_{mean,L}-{T}_{U}$ is the difference between the mean temperatures of both plates, and ${\theta }_{p}$ is the difference between the maximum and minimum of the periodic temperature component along the lower plate. The heating and topography patterns are characterized by the same wave number $\alpha$, and $\Omega$ is the phase difference measuring the relative position of the temperature and groove patterns. Half of the mean slot height *h* is used as the length scale, $\kappa \nu /(g\Gamma {h}^{3})$ is used as the temperature scale, $k$ denotes the thermal conductivity, *c* stands for the specific heat, $\kappa =k/\rho c$ is the thermal diffusivity, $\nu$ is the kinematic viscosity, $\mu$ denotes the dynamic viscosity, $g$ denotes the gravitational acceleration and $\Gamma$ stands for the thermal expansion coefficient. The motion of the Boussinesq fluid in the slot is described by the field equations of the form

3a $$\frac{\partial u}{\partial x}+\frac{\partial v}{\partial y}=0,$$

3b $$u\frac{\partial u}{\partial x}+v\frac{\partial u}{\partial y}=-\frac{\partial p}{\partial x}+{\nabla }^{2}u,$$

3c $$u\frac{\partial v}{\partial x}+v\frac{\partial v}{\partial y}=-\frac{\partial p}{\partial y}+{\nabla }^{2}v+{Pr}^{-1}\theta ,$$

3d $$u\frac{\partial \theta }{\partial x}+v\frac{\partial \theta }{\partial y}={Pr}^{-1}{\nabla }^{2}\theta$$

where ($u$, $v$) are the velocity components in the (*x*, *y*) directions, respectively, scaled with ${U}_{v}=\nu /h$ as the velocity scale, $p$ stands for the pressure scaled with $\rho {U}_{v}^{2}$ as the pressure scale, *Pr* = $\nu /\kappa$ is the Prandtl number, and the gravity acts in the negative *y*-direction. The relevant boundary conditions have the form

4 $$u\left(x,{y}_{L}\right)=u\left(x,1\right)=0, \quad v\left({x,y}_{L}\right)=v\left(x,1\right)=0, \quad \theta \left(x,{y}_{L}\right)=\theta{}_{L}\left(x\right),\theta (x,1)=0.$$

The presence of any horizontal mean pressure gradient is excluded by imposing a constraint of the form

5 $$\left. {\frac{{\partial p}}{{\partial x}}} \right|_{{mean}} = 0.$$

Problems (1)–(5) has been solved numerically with spectral accuracy using Fourier expansions in the *x*-direction, Chebyshev expansions in the *y*-direction and the spectrally accurate Immersed Boundary Conditions (IBC) method ^5,6^ to handle the boundary irregularities. Experimental plate temperatures were used in the computations.

Case *A*, which involves two isothermal plates with a grooved lower plate, has geometry corresponding to $A=0.1$, $\alpha =1$ and thermal field characterized by ${Ra}_{p}=0,{Ra}_{uni}=210$. Case *B*, which involves two smooth plates with the lower plate exposed to a periodic heating, has geometry corresponding to $A=0$ and thermal field characterized by ${Ra}_{p}=1500$ and ${Ra}_{uni}=-100$, with the latter accounting for the difference between the upper plate temperature and the mean temperature of the lower plate encountered in the experiment. The controls available in the experimental apparatus did not provide exact matching of the mean temperatures of both plates. Cases *C1* and *C2*, which involve the upper smooth plate and the lower corrugated plate exposed to periodic heating with the same period as the groove period, correspond to $A=0.1$ defining the geometry, ${Ra}_{p}=1500$ defining the intensity of periodic heating and ${Ra}_{uni}=-30$ accounting for the mismatch between the upper plate temperature and the mean temperature of the lower plate encountered in the experiment. This is the most general case where the system response is dictated by the pattern interaction effect.

## Methodology—experiment

**Figure 6:**
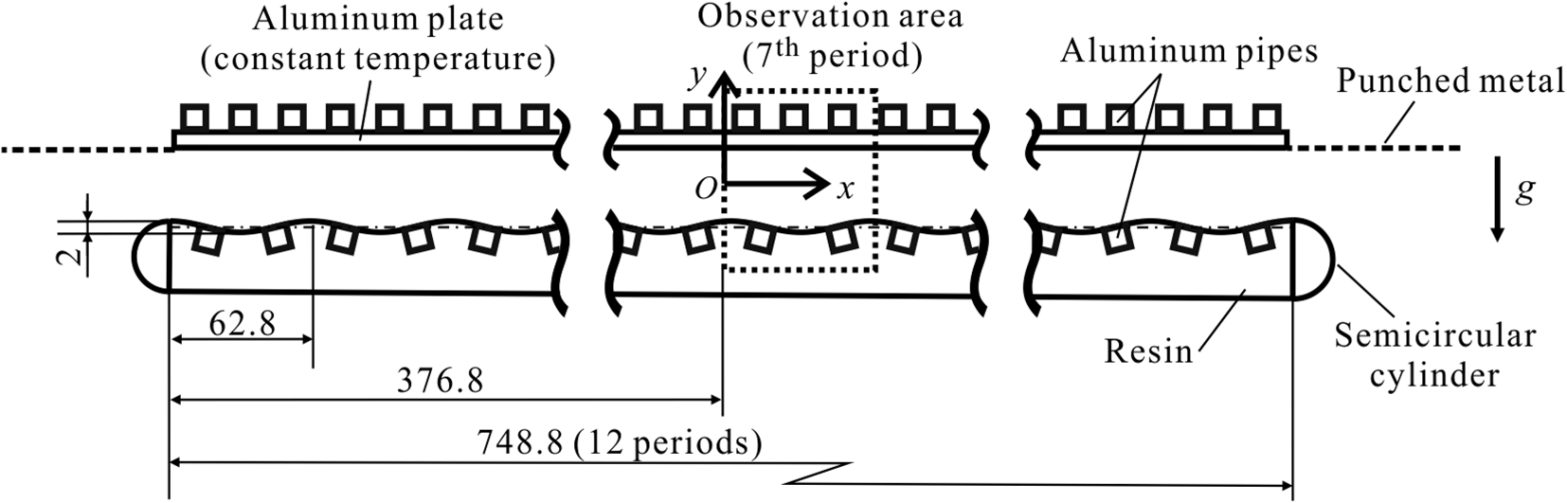
Sketch of the experimental apparatus (all dimensions are in mm) used for experiments *C*1 and *C*2. Both ends of the apparatus were closed in the experiments *A* and *B*. The upper plate remained the same in all experiments. The lower plate was replaced with an isothermal grooved plate for experiment *A* and with a smooth plate subject to periodic heating for experiment *B*. See text for details.

A sketch of the experimental apparatus using air as the working fluid is shown in Fig. 6. The upper smooth plate is made of a 5 mm-thick aluminum plate whose temperature is controlled using tubes with cooling liquid.

Three types of experiments were carried out. Experiment *A* was used to demonstrate the system response when exposed to a spatially distributed stimulus in the form of periodic grooves. The lower plate was made of an ABS resin bar with sinusoidal grooves of wavelength ${\lambda }^{*}=62.8$ mm and amplitude A^*^ = 1 mm manufactured using CNC machining. A 0.3 mm-thick Copper plate was glued to realize uniform heating of the grooves. The plate was kept isothermal using embedded tubes with heated liquid with controlled temperature. The ends of the slot were closed. Experiment *B* was used to demonstrate the system response when subject to a stimulus in the form of spatially periodic heating. The lower plate was made of an ABS resin covered with a 0.2 mm-thick waterproof paper to realize a horizontal temperature gradient. Two-tube system carrying cooling and heating liquids created periodic temperature variations. The slot had its end closed. Experiments *C1* and *C2* were used to demonstrate the system response to a combination of two different stimuli. The lower plate was equipped with sinusoidal grooves and with two-tube system carrying cooling and heating liquids providing the means for creation of periodic variations of temperature along its surface. The surface was covered with water-proof paper as in experiment *B*. The slot ends were open to provide the means for the global system response. The wavelength of temperature variations was the same as the groove wavelength, providing perfect tuning between both stimuli. A change in the relative position of the heating pattern with respect to the groove patterns was produced by switching pipe connections, providing the means for creation of two different phase shifts, i.e., $\Omega =\pi /2$ (experiment *C1*) and $\Omega =-\pi /2$ (experiment *C2*).

The mean slot opening in all experiments was 2h^*^ = 20 mm. The slot width was 400 mm which produced the aspect ratio 20. The horizontal length of the slot was 748.8 mm for all the experiments. The groove and heating wavenumber were $\alpha =1$. The groove amplitude was ${A}^{*}=1$ mm. The temperature of the upper plate was ${T}_{U}=20.0$ °C, the temperature of the lower plate was ${T}_{L}=22.4$ °C for experiment *A*, the mean temperature of the lower plate for experiments *B*, *C1* and *C2* were $19.0$ °C, $19.7$ °C and $19.7$ °C, respectively, and the amplitude of temperature variations (peak-to-peak value) in these cases was 15.0 °C. The surface temperature distributions were measured using an Infrared Thermal Imaging Camera (Nippon Avionics H2640). During experiments, the surface temperature was monitored using sheet form thermocouples (Chino C060, Type T). A laser light sheet with a thickness of approximately 1 mm was used to illuminate $1$-μm-diameter seeding in the (*x, y*)-plane. The length of the conduit resulted from the use of twelve stimuli wavelengths, with most of the observations made around the 7th wavelength. The temperature of the lower surface in experiment *A* and the upper surface for all the experiments was uniform with variation less than $\pm 0.2^\circ \mathrm{C}$ except at the periphery of the plate. The two-dimensionality of the *x*-periodic temperature of the lower plate in experiments *B*, *C1* and *C2* was verified: the spanwise variations of hot-spot temperature were less than $\pm 0.5$ °C. The two-dimensionality of the flow fields was verified by measuring velocity at different spanwise locations using particle image velocimetry (PIV).

## Supplementary Information

Supplementary Legends.

Supplementary Video A.

Supplementary Video B.

Supplementary Video C1.

Supplementary Video C2.
