# Supplementary material for: Pattern interaction effect
Source: Sci Rep. 2021 Jul 16;11:14573. doi: 10.1038/s41598-021-93707-6 (PMC8285401; doi:10.1038/s41598-021-93707-6)
Supplement: Supplementary file 1 — Supplementary Legends. [file 41598_2021_93707_MOESM1_ESM.pdf]

Pattern Interaction Effect

J.M. Floryan [1] *and A. Inasawa* [2]

[1] *Professor, Department of Mechanical and Materials Engineering*

The University of Western Ontario, London, Ontario, Canada, N6A 5B9, floryan@uwo.ca

[2] *Associate Professor, Department of Aeronautics and Astronautics*

Tokyo Metropolitan University, Asahigaoka 6-6, Hino, Tokyo 191-0065, Japan

Guide for supplementary material

Movie *A*: Two isothermal plates. The upper plate is smooth, the lower plate has sinusoidal grooves with the amplitude $A = 0.1$ and the wave number $\alpha = 1$. The heating conditions correspond to the uniform Rayleigh number $Ra_{uni} = 210$. The periodic Rayleigh number is $Ra_p = 0$ as both plates are isothermal.

Movie *B*: Two smooth plates. The upper plate is isothermal. The lower plate is exposed to a periodic heating with intensity corresponding to the periodic Rayleigh number $Ra_p = 1500$ and the spatial distribution corresponding to the wave number $\alpha = 1$. The difference between the mean temperatures of the plates corresponds to the uniform Rayleigh number $Ra_{uni} = -100$.

Movie *C1*: The upper plate is smooth and isothermal. The lower plate has grooves with the amplitude $A = 0.1$ and the wave number $\alpha = 1$. This plate is exposed to a periodic heating with the wave number $\alpha = 1$ with its intensity corresponding to the periodic Rayleigh number $Ra_p = 1500$. The difference between the mean temperatures of the plates corresponds to the uniform Rayleigh number $Ra_{uni} = -30$. The phase difference between the heating and groove patterns corresponds to $\Omega = \pi/2$.

Movie *C2*: All conditions are the same as in Movie *C1* except for the phase difference, which corresponds to $\Omega = -\pi/2$.
